# Psychobiological effects of an eHealth psychoeducational intervention to informal caregivers of persons with dementia: a pilot study during the COVID-19 pandemic in Italy

**DOI:** 10.1007/s40520-023-02610-9

**Published:** 2023-11-09

**Authors:** Claudio Singh Solorzano, Nadia Cattane, Anna Mega, Stefania Orini, Orazio Zanetti, Rabih Chattat, Moira Marizzoni, Michela Pievani, Annamaria Cattaneo, Cristina Festari

**Affiliations:** 1 Laboratory of Alzheimer’s Neuroimaging and Epidemiology, IRCCS Istituto Centro San Giovanni di Dio Fatebenefratelli, Via Pilastroni 4, 25125 Brescia, Italy; 2 Biological Psychiatry Unit, IRCCS Istituto Centro San Giovanni di Dio Fatebenefratelli, Brescia, Italy; 3 UOC Neurologia, ULSS 9 Scaligera-Distretto 4, Verona, Italy; 4 Alzheimer Unit, IRCCS Istituto Centro San Giovanni di Dio Fatebenefratelli, Brescia, Italy; 5 Department of Clinical and Experimental Sciences, Università degli Studi di Brescia, Brescia, Italy; 6 Department of Psychology, Università di Bologna, Bologna, Italy; 7 Department of Pharmacological and Biomolecular Sciences, University of Milan, Milan, Italy

**Keywords:** Caregivers, Dementia, Psychoeducation, Caregiver burden, Self-efficacy, Cortisol

## Abstract

**Background:**

The workload associated with caring for a person with dementia (PwD) could negatively affect informal caregivers’ physical and mental health. According to the recent literature, there is a need for studies testing the implementation of affordable and accessible interventions for improving caregivers’ well-being.

**Aims:**

This study aimed to explore the feasibility and effectiveness of an 8 week eHealth psychoeducation intervention held during the COVID-19 pandemic in Italy in reducing the psychological burden and neuroendocrine markers of stress in caregivers of PwD.

**Methods:**

Forty-one informal caregivers of PwD completed the eHealth psychoeducation intervention. Self-reported (i.e., caregiver burden, anxiety symptoms, depressive symptoms, and caregiver self-efficacy) and cortisol measurements were collected before and after the intervention.

**Results:**

Following the intervention, the caregivers’ self-efficacy regarding the ability to respond to disruptive behaviours improved (*t* = − 2.817, *p* = 0.007), anxiety and burden levels decreased (state anxiety: *t* = 3.170, *p* = 0.003; trait anxiety: *t* = 2.327, *p* = 0.025; caregiver burden: *t* = 2.290, *p* = 0.027), while depressive symptoms and cortisol levels did not change significantly. Correlation analyses showed that the increase in self-efficacy was positively associated with the improvement of caregiver burden from pre- to post-intervention (*r* = 0.386, *p* = 0.014). The intervention had a low rate of dropout (*n* = 1, due to the patient’s death) and high levels of appreciation.

**Discussion:**

The positive evidence and participation rate support the feasibility and effectiveness of the proposed eHealth psychoeducational intervention to meet the need for knowledge of disease management and possibly reduce detrimental effects on caregivers’ psychological well-being.

**Conclusion:**

Further placebo-controlled trials are needed to test the generalizability and specificity of our results.

**Supplementary Information:**

The online version contains supplementary material available at 10.1007/s40520-023-02610-9.

## Introduction

Approximately 55.2 million adults worldwide are estimated to live with dementia, and these numbers are expected to grow to 139 million by 2050 [1]. Family members or friends of a person with dementia (PwD) often provide some type of unpaid assistance (e.g., in activities of daily living, emotional support, supervision, etc.), assuming the role of the informal caregiver. Caring for a loved one with dementia is often complex and time-consuming and could affect different domains of caregiver functioning. Indeed, caring for a PwD could affect caregiver’s stress levels, leading to negative consequences for their physical and mental health, financial savings, productivity, and psychological well-being [2]. Being a caregiver could represent a factor of chronic stress [3] and is associated with a higher likelihood of chronic diseases, impaired hypothalamic–pituitary–adrenal (HPA) axis functioning, and compromised immune response [4, 5]. Cortisol is the primary marker of HPA-axis functioning, and its dysregulation is thought to play a crucial role in the aetiology of several physical diseases and mental disorders [6–8]. The long-term stressful condition of caregivers of PwD increases aberrant and dysfunctional changes in the HPA-axis activity and cortisol secretion, supporting the biological pathway through which stress contributes to adverse physical and mental health outcomes [4].

In the last years, in part due to the WHO’s commitment towards dementia families [1], growing evidence has shown that supporting informal caregivers with non-pharmacological interventions benefits both the caregiver and the PwD by enhancing their quality of life and care and enabling them to stay at home for a longer period of time [9]. A recent meta-analysis on the effectiveness of non-pharmacological interventions on psychological health and quality of life in caregivers of PwD reported that psychoeducation was effective in reducing depressive symptoms and caregiver burden and the only effective intervention to improve anxiety symptoms [10].

Due to technological development and the COVID-19 pandemic, in the last years, Internet-based and remote interventions (i.e., eHealth interventions) have become popular and widely used to support and help caregivers of PwD to deal with emotional and practical difficulties in their assistance activities [11]. The eHealth interventions have the potential to overcome many barriers to help-seeking, are less costly, easier to access and more combinable with other work or family tasks [12]. Moreover, meta-analyses showed that eHealth psychoeducation interventions have beneficial effects on caregivers’ mental health and well-being as “traditional” in-person interventions [13, 14]. However, the high heterogeneity in the design of eHealth interventions, such as caregiver characteristics, intervention length, theoretical foundation, or web-based mode of delivery, makes it difficult to compare the effectiveness of Internet-based interventions [13–15]. During the recent worldwide COVID-19 pandemic, caregiver stress levels and emotional burden increased due to the social and emotional challenges of the period [2, 16, 17]. However, despite a few studies showing the beneficial value of e-health psychoeducation interventions on caregivers of PwD during the COVID-19 pandemic [18, 19], there was very scarce evidence in the Italian context.

Aim 1: This study aimed to explore the effectiveness of an eHealth psychoeducation intervention held during the COVID-19 pandemic in the Italian context in reducing psychological and cortisol levels in caregivers of PwD. The intervention’s effectiveness was evaluated by measuring anxiety, depression, caregiver burden, and self-efficacy. We also collected salivary samples to measure an objective biomarker of stress by quantifying cortisol levels. Since the intervention primarily focused on providing practical information to respond to daily challenges related to caring for a PwD, we expected caregivers to improve their levels of anxiety, depression, burden, and self-efficacy, and reduce the cortisol output over a day from pre- to post-intervention.

Aim 2: In addition, we explore the role of the change in self-efficacy due to the eHealth psychoeducation intervention on improving psychobiological well-being. We hypothesised that a significant increase in the self-efficacy dimensions (i.e., the main target of our intervention) could be associated with a greater improvement in the psychological and neuroendocrine markers that significantly change from pre- to post-intervention.

## Methods

This study was conducted during the second and third waves of the COVID-19 pandemic in Italy. The first wave occurred from March to June 2020, the second wave from September to December 2020, and the third one from January to mid-2021. In the second and third waves, regions and provinces of Italy were classified into three areas according to their specific epidemiological risk scenario: red, orange, and yellow. Data collection occurred in a red zone (i.e., the highest risk of classification), with high individual movement restrictions and COVID-19 containment measures. The study was approved by the IRCCS Fatebenefratelli Ethics Committee (approval date July 17, 2020; Number 38/2020), and informed consent was obtained from all participants.

### Study design and participants

In this single-arm pre–post-study design, 42 informal caregivers of community-dwelling persons with mild-to-moderated dementia participate in an eHealth psychoeducation intervention. The severity of the cognitive impairment of the PwD was evaluated using the score of the Mini-Mental State Examination (MMSE), and caregivers of PwD with the MMSE score range between 10 and 23 were included [20]. Participants had to meet the following inclusion criteria: they were caregivers for at least 6 months, provided care for at least 2 h per day to their one-loved, had to have a connected device, and were Italian native speakers. Exclusion criteria were the presence of a current psychiatric illness or metabolic disease. This information was collected by a trained psychologist in an individual telephone interview using the Cumulative Illness Rating Scale [21]. Before and after the intervention, participants completed clinical questionnaires, provided saliva samples, and, at the end of the intervention, answered satisfaction questions. Clinical questionnaires before and after the intervention and satisfaction questions after the intervention were collected remotely using Google Forms.

### eHealth psychoeducational intervention

The psychoeducational intervention consisted of eight weekly 2 h sessions and was based on the model of the ‘Savvy Caregiver Program’ [22] and the ‘Medway Carers Courses’ [23]. In particular, the duration and topics of the intervention (Table 1S, Supplementary Material), the length of each session, and the presence of invited expert speakers in the sessions were mainly based on the ‘Medway Carers Courses’. We slightly reduced the number of sessions compared to the original model (from 10 to 8) to make it more suitable for an online and remote intervention setting. In addition, the well-known teaching framework of the ‘Savvy Caregiver Program’ was applied to increase caregivers’ knowledge of dementia and its consequences on different aspects of patients’ life (e.g., cognitive, emotional, behavioural, etc.). This method allows the caregiver to flexibly and adaptively deal with caring difficulties considering the results of the applied solution strategies to daily problems and the feedback of the patient [22, 24]. The choice of these two models was based on their excellent feasibility, efficacy, and ease of implementation in an eHealth setting. Therefore, grounded on these two models, we developed a multi-perspective intervention that allowed caregivers to feel high levels of self-confidence, sense of control, and self-efficacy, providing the best strategies to deal with the challenging daily situations of dementia care. Indeed, the main aims of the intervention were to provide caregivers with the practical knowledge, skills, and attitudes needed to carry out their assistance role (e.g., managing patients’ cognitive and behavioural symptoms, creating a prosthetic environment at home, obtaining information on disease progression, available treatments, relief services and legal issues), raise awareness of their emotional needs, and create a long-lasting local support network. In each session, an invited speaker expert in specific aspects of dementia (i.e., a geriatrician, a neuropsychologist, a social worker, a lawyer, an occupational therapist, or speakers from local support organisations) introduced a specific topic (Table 1S, Supplementary Material). The intervention was carried out online, synchronously, and in small groups of 10–12 persons to encourage engagement and reciprocal sharing. The intervention was led by a psychotherapist expert in dementia care (AM) who was present throughout, introduced the speakers, and facilitated the discussion.

**Table 1:** Characteristics of caregivers and PwD (*N* = 41)

Features of caregivers of PwD	M ± SD	*N* (%)
Age	57.15 ± 10.58	
Sex—female		32 (78.0)
Education level		
Middle school and below		18 (43.9)
High school		14 (34.1)
University and above		9 (22.0)
Relationship with PwD		
Spouse		12 (29.3)
Child		25 (61.0)
Other relatives		4 (9.8)
Living with PwD—yes		21 (51.2)
Caregiver time spent on ADLs (hours per day)	1.44 ± 1.90	
Caregiver time spent on IADLs (hours per day)	3.07 ± 1.81	
Caregiver time spent on supervision (hours per day)	1.68 ± 4.45	
Features of PwD		
Age	77.61 ± 7.94	
Sex—female		25 (61.0)
NPI	17.88 ± 11.40	
Diagnosis		
Alzheimer’s disease		26 (63.4)
Vascular dementia		5 (12.2)
Dementia with Lewy bodies		4 (9.8)
Other aetiologies		6 (14.6)

*PwD* person with dementia; *ADLs* Personal activities of daily living; *IADLs* Instrumental activities of daily living; *NPI* Neuropsychological Inventory

### Sociodemographic features

During an individual telephone interview before the start of the intervention, a trained psychologist collected participants’ sociodemographic variables (i.e., age, gender, and education levels) and information on their role as caregivers (i.e., relationship with PwD, living arrangement, and time spent at day assisting and supervising the PwD). Daily care engagement was measured using a subscale of the Resource Utilization in Dementia (RUD) instrument [25] and was operationalised as the total amount of hours in a typical day spent in the past month on the activities of daily living (ADL), instrumental activities of daily living (IADL), and surveillance. In addition, in an individual telephone interview with the caregiver, an expert neuropsychologist collected sociodemographic and clinical details of the patient, including diagnosed dementia subtype. In the same telephone interview, the severity of the patient’s neuropsychiatric symptoms was assessed using the Neuropsychiatric Inventory (NPI) questionnaire [26]. The NPI is a 12-item informant-based interview that collects the frequency and severity of neuropsychiatric symptoms (i.e., delusions, hallucinations, agitation/aggression, depression, anxiety, euphoria/elation, apathy/indifference, disinhibition, irritability/lability, aberrant motor behaviours, sleep disturbance/night-time behaviours, and eating problems) over the previous month. The maximum total score is 144, with higher scores indicating greater behavioural and psychological disturbances.

### Clinical assessments

#### Caregiver burden

Caregiver burden was measured using the Italian version of the Zarit Burden Interview (ZBI) [27, 28], a 22-item self-reported questionnaire widely used to evaluate the subjective stress related to the specific caregiver role. Each item is rated on a 5-point Likert scale that ranges from 0 (i.e., never) to 4 (i.e., nearly always). The total burden score was obtained by the sum of all items and ranged between 0 and 88, with higher scores indicating a higher burden of care. The ZBI was extensively used in informal caregivers of PwD [29, 30], showing good internal consistency and reliability [27]. The questionnaire was administered before and after the intervention using Google Forms. At pre-intervention assessment, the Cronbach’s α of the questionnaire in this study was 0.91 and McDonald’s ω was 0.90. At post-intervention assessment, the Cronbach’s α of the questionnaire in this study was 0.91 and McDonald’s ω was 0.91.

#### Anxiety symptoms

The Italian version of the State-Trait Anxiety Inventory (STAI) was used to measure state and trait anxiety [31, 32]. The STAI was composed of two subscales: the state anxiety scale (STAI-Y1) and the trait anxiety scale (STAI-Y2). The STAI-Y1 refers to the transitory and momentary anxiety experience when the respondent compiled the scale, whereas the STAI-Y2 was related to the general and stable proneness to experience anxiety [33]. Each subscale consisted of 20 items scored on a 4-point Likert scale ranging from 1 (i.e., not at all) to 4 (i.e., very much so). The total score of each scale ranged from 20 to 80, with higher scores reflecting greater anxiety symptoms. The STAI was used in the previous studies on caregivers of PwD, showing good internal consistency [34]. The questionnaire was administered before and after the intervention using Google Forms. In the present sample, internal consistency of the subscales was excellent in both pre-assessment (Cronbach’s α of STAI-Y1 = 0.94, McDonald’s ω of STAI-Y1 = 0.93, Cronbach’s α of STAI-Y2 = 0.91, and McDonald’s ω of STAI-Y2 = 0.91) and post-assessment (Cronbach’s α of STAI-Y1 = 0.94, McDonald’s ω of STAI-Y1 = 0.94, Cronbach’s α of STAI-Y2 = 0.91, and McDonald’s ω of STAI-Y2 = 0.90).

#### Depressive symptoms

The Italian version of the Beck Depression Inventory-II (BDI-II) was used to assess the levels of depressive symptoms in the previous two weeks [35, 36]. It is a 21-item self-report scale, and responses to each statement are scored on a 4-point Likert scale ranging from 0 (i.e., the absence of the symptom) to 3 (i.e., the severe or persistent presence of the symptom). The sum of all items ranges from 0 to 63, with higher scores indicating higher levels of depressive symptoms. The BDI-II has previously been administered in studies of caregivers of PwD [37]. The questionnaire was administered before and after the intervention using Google Forms. In the current study, the Cronbach’s alpha was 0.92 and the McDonald ω was 0.92 at pre-intervention assessment, whereas the Cronbach’s alpha was 0.91 and the McDonald ω was 0.91 at post-intervention assessment.

#### Self-efficacy

Caregiver level of self-efficacy (i.e., an individual’s belief that they succeed in a specific situation or can accomplish a specific task) was measured using the Italian version of the Revised Scale for Caregiving Self-efficacy (RSCSE) [38, 39]. The 15 items of RSCSE evaluated the perceived capacity of caregivers to deal with the challenges of dementia caregiving (Steffen et al., 2019). Three different 5-item subscales were obtained from the RSCSE: self-efficacy in obtaining respite subscale (SE-OR), self-efficacy in responding to disruptive behaviours subscale (SE-RDB), and self-efficacy in controlling upsetting thoughts (SE-CUT). In particular, the SE-OR evaluated the caregivers’ confidence to ask for help and obtain rest from their caregiving tasks. The SE-RDB assessed the perceived ability of caregivers to manage their emotions when they deal with memory or behavioural problems of care recipients. Finally, the SE-CUT measured the caregivers’ ability to control or restrict distressing and negative thoughts about caregiving due to their sacrifices and the burden associated with their assistance role [40]. Each item is scored from 0 to 100%, with high scores indicating high self-efficacy. For each subscale, an average total score was obtained as in other studies on caregivers of PwD [41, 42]. The questionnaire was administered before and after the intervention using Google Forms. Cronbach’s α of the RSCSE subscales in this study ranged from 0.91 to 0.96 at pre-intervention and from 0.93 to 0.96 at post-intervention, whereas McDonald’s ω ranged from 0.91 to 0.96 at pre-intervention and from 0.93 to 0.96 at post-intervention.

### Cortisol assessment

The saliva samples were collected using Salivettes© (Sarstedt, Leicester, UK). On average, a week before the start of the intervention, the collection method was explained, an instruction sheet was provided, and a pack containing eight labelled Salivette tubes was given to each participant. Briefly, participants were instructed to place the Salivette© cotton swab in their mouth and chew it for 1 min. Saliva samples were obtained before (about 1 day) and after (about 5 days) the intervention. Participants provide four saliva samples for each day at set time points: on awakening, 30 min after awakening, at noon, and at around 7 pm. Participants were told not to eat, drink, or smoke cigarettes prior to giving each sample. They were also required to complete a table as a record of their sampling schedule. Saliva samples were stored immediately in participants’ home freezers before being collected and transported on ice by the researcher to the analysis laboratory.

Prior to the analysis of the cortisol concentration, samples were thawed completely for at least 2 h and centrifuged at 1500 × *g* for 15 min to remove debris, including mucins and other particulates that may interfere with antibody binding, from the saliva. High-sensitivity salivary cortisol enzyme immunoassay (EIA) kits (No. 1-3002-5 Salimetrics LLC, PA, USA) were used to determine cortisol levels in participant samples. This is a competitive immunoassay kit. The intra- and inter-assay variability of the cortisol kit was 5.96% and 9.44%, respectively, and the assay’s detection limit was between 3.000 and 0.012 ug/dL, according to the highest and lowest standard of the curve, respectively. Optical density measurements were performed on a standard plate reader at 450 nm. Concentrations of the selected compounds were calculated using KC4 v 3.4 software Rev 21 (Bio-Tek instruments). The overall volume of cortisol released over the day was computed by trapezoidal calculation of all the collected samples (i.e., at awakening, 30 min after the awakening, at noon, and at 7 pm) [43].

### Satisfaction survey

At the end of the intervention, participants anonymously answered an ad hoc survey created on Google Forms to gauge their perceptions of the online intervention’s quality, usefulness, and feasibility. In particular, caregivers rated their satisfaction with i) each session topic and the presentation way, using a Likert 5-point scale and ii) the online modality delivery of the course, using a binary scale (i.e., yes/no). Finally, we asked caregivers to indicate the material they considered more valuable and practically useful to their caregiving role from a list of key points discussed over the course.

### Statistical analyses

All data analyses were conducted using IBM SPSS Statistic version 28 (SPSS Inc., Armonk, NY, USA). Due to the pilot nature of the study, no formal sample size calculations were conducted. However, to ensure a suitably reliable estimate of the standard deviations to power a future trial with 90% power, at least 25 people were recommended if the expected effect size was between 0.1 and 0.3 [44]. Based on previous meta-analysis results of the effects of eHealth interventions on caregiver burden levels [12], we expect an effect size of 0.13. As we assume a dropout rate of 16% [45], a minimum target sample size of 29 was adopted. Cronbach’s alpha (*α*) and McDonald’s omega (*ω*) were computed for all the scales used to assess their reliability in this study [46]. Descriptive data of demographic, psychological, and cortisol variables were reported as mean (M) supplemented by the standard deviation (SD) or as the number of participants (N) with the percentage in parenthesis. The satisfaction survey results were reported as median (Mdn) and interquartile range (IQR) or as the percentage of participants. The pre- and post-intervention cortisol AUC_g_ was naturally logarithmically transformed before statistical analyses to normalise its distribution. Paired *t* tests were used to explore the change in psychological and cortisol variables from the pre- to post-treatment. The effect sizes (i.e., Cohen’s *d*) for all these comparisons were reported. Cohen’s *d* > 0.20 was considered a small effect, > 0.50 a medium effect, and > 0.80 a large effect [47]. We calculated a change score for significantly changed variables from pre- to post-intervention. We subtracted the post-intervention score from the pre-intervention score for caregiver burden, state anxiety, trait anxiety, depressive symptoms, and cortisol concentration. Conversely, we subtracted pre-intervention scores from post-intervention scores for all self-efficacy subscales. In this way, all change scores are expressed as improvements in the measured variable. Partial correlations adjusting for the NPI score were computed to assess the relationship between the changes scores. We controlled for the behavioural and psychological disturbances factor, since it is well-known relationship with the change in caregivers’ self-efficacy and emotional burden [48, 49]. Two-tailed tests were used throughout, and the significance level was set at *p* < 0.05.

## Results

### Analytic sample

**Fig. 1:**
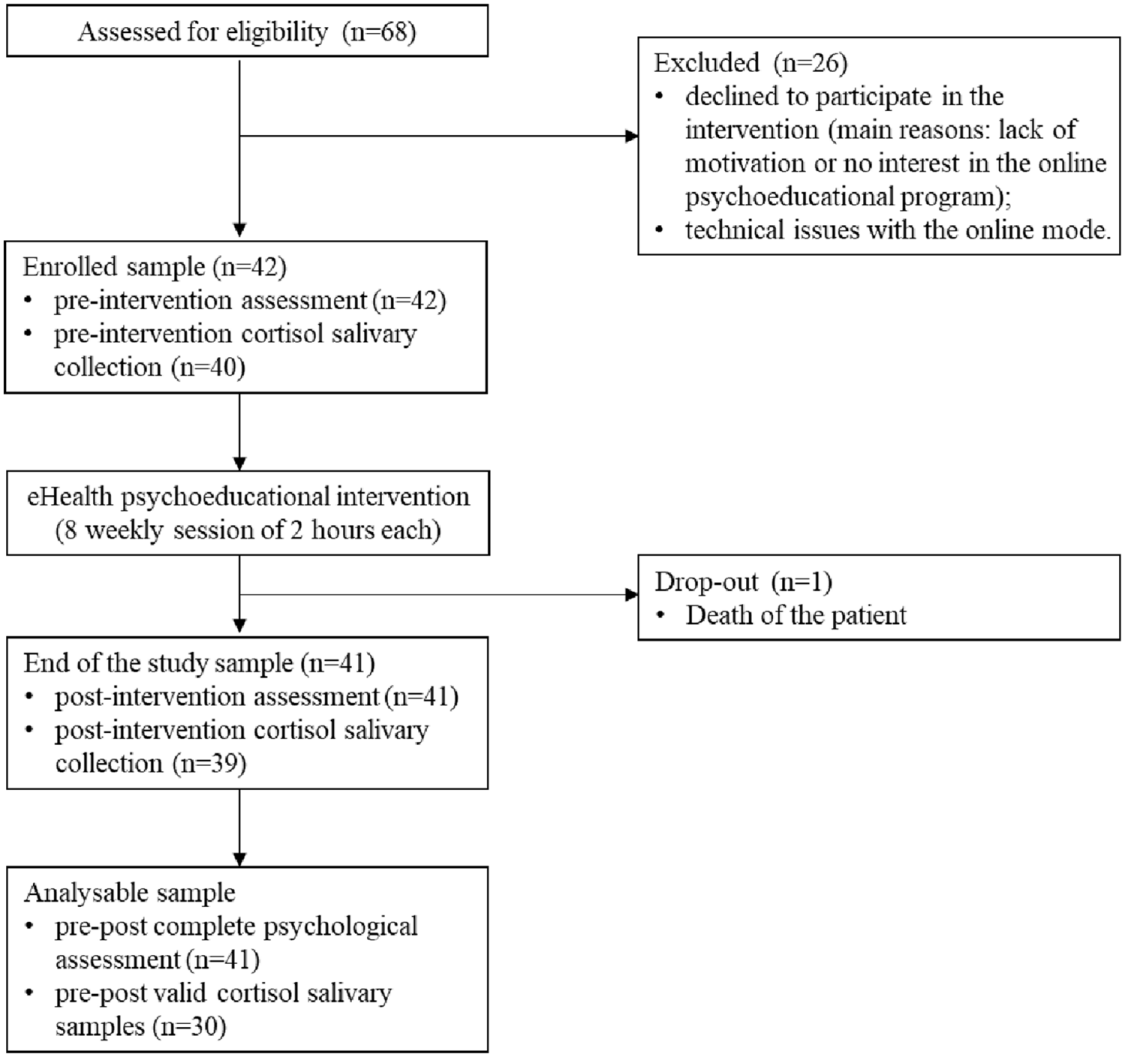
Recruitment flowchart

The recruitment flowchart is shown in Fig. 1. Caregivers were recruited through contacts with the social services of two Italian municipalities near Brescia, Italy (i.e., Roncadelle and Castel Mella; *n* = 44), the local memory clinic (*n* = 13), or through a newspaper advertisement (*n* = 11). Of the total eligible sample (*n* = 68), 26 caregivers declined to participate in the intervention, mentioning a lack of motivation, no interest in the eHealth psychoeducational program, or technical issues with the online mode. Of the 42 enrolled caregivers, only a participant dropped from the study due to the patient’s death, leaving an analytic sample of 41 caregivers of PwD. Eleven participants provided insufficient saliva samples (i.e., samples did not contain a sufficient amount of saliva on which to perform the analyses), confusing saliva samples (i.e., samples collected outside the schedule) or did not provide the saliva samples. Therefore, cortisol analyses were computed on a smaller sample size (*N* = 30).

### Baseline characteristics of caregivers and patients with dementia

Table 1 summarises the descriptive characteristics of the 41 caregivers and their loved ones who completed this study. The NPI scores ranged from 0 to 46, with the highest scores for eating problems (*M* = 3.07; SD = 4.06), apathy/indifference symptoms (*M* = 2.49; SD = 3.49), and sleep disturbances (M = 2.19; SD = 3.68). 75.6% of caregivers attended all the course sessions (Mdn = 8; IQR = 1).

### Changes across time: psychological and biological outcomes

Table 2 shows the comparison between the different variables of the study before and after the intervention.

**Table 2:** Descriptive statistics and comparison over time for the self-reported and biological variables of caregivers of PwD (*N* = 41)

Variable	Pre-intervention	Post-intervention	Change scores	*t* (df)	*p* value	Cohen’s *d*
	*M* ± *SD*	*M* ± *SD*	*M* ± *SD*			
Caregiver burden	38.00 ± 16.06	33.71 ± 14.21	4.29 ± 12.00	2.290 (40)	**0.027**	0.358
State anxiety	48.66 ± 11.69	44.05 ± 11.04	4.61 ± 9.31	3.170 (40)	**0.003**	0.495
Trait anxiety	44.61 ± 10.33	42.12 ± 9.69	2.49 ± 6.84	2.327 (40)	**0.025**	0.363
Depressive symptoms	11.68 ± 9.74	10.51 ± 9.14	1.17 ± 6.44	1.164 (40)	0.251	0.182
Self-efficacy—OR	54.10 ± 28.66	59.05 ± 26.69	4.95 ± 25.09	− 1.263 (40)	0.214	0.197
Self-efficacy—RDB	55.91 ± 25.78	66.00 ± 20.05	10.09 ± 22.93	− 2.817 (40)	**0.007**	0.440
Self-efficacy—CUT	57.03 ± 22.90	59.76 ± 22.40	2.72 ± 23.60	− 0.739 (40)	0.464	0.115
Cortisol AUC_g_ (nmol/l*min)^	8.08 ± 0.54	8.05 ± 0.44	0.03 ± 0.51	0.321 (29)	0.751	0.059

Bold indicates *p* < 0.05

^*N* = 30. Self-efficacy—OR: self-efficacy in obtaining respite subscale; Self-efficacy—RDB: self-efficacy in responding to disruptive behaviours subscale; Self-efficacy—CUT: self-efficacy in controlling upsetting thoughts; Cortisol AUC_g_: natural logarithm of cortisol area under the curve with respect to the ground

**Fig. 2:**
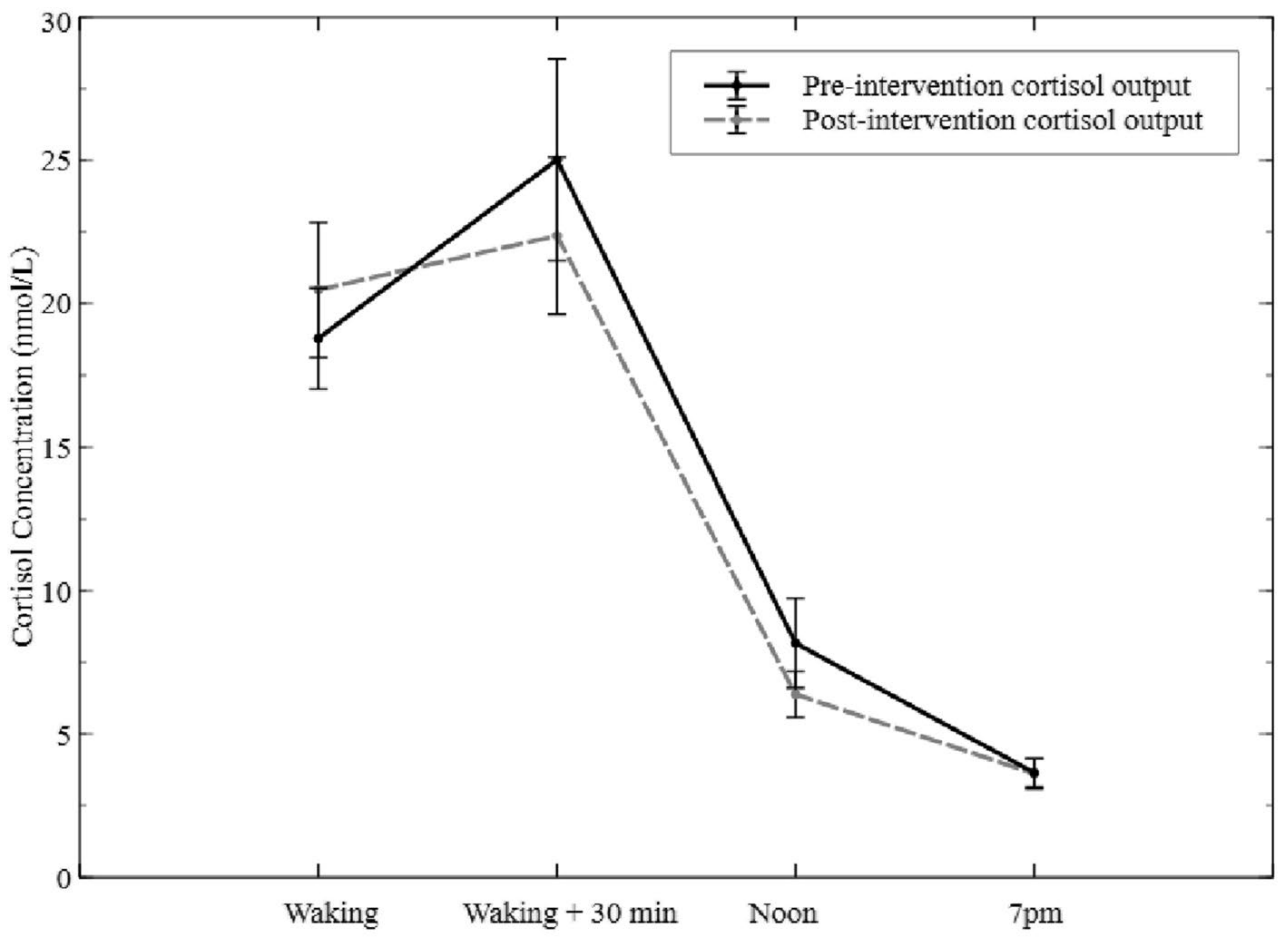
Diurnal cortisol output of caregiver at pre-intervention and post-intervention. Bars indicate standard errors of the mean (*N* = 30)

Raw data of cortisol output over the day (i.e., cortisol AUC_g_) were slightly higher before the intervention (mean = 3779.97 nmol/l*min, SD = 2671.60 nmol/L) compared with post-intervention (mean = 3474.46 nmol/L*min, SD = 1802.53 nmol/L). However, this change was non-significant (Fig. 2).

### Role of change in self-efficacy

**Fig. 3:**
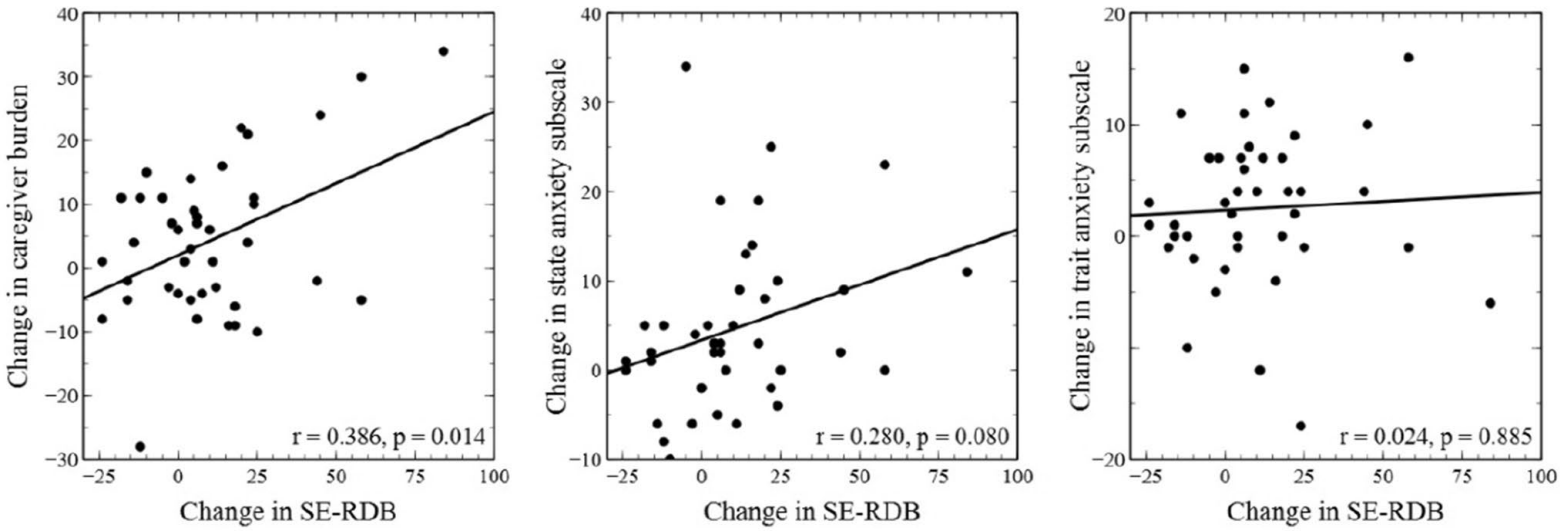
Correlation scatterplots of self-efficacy in responding to disruptive behaviours subscale and caregiver anxiety/burden scores (*N* = 41). *SE-RDB* self-efficacy in responding to disruptive behaviours subscale. All changes scores are expressed as improvements of the measured variable. The values in the graphs denote correlation coefficient and associated *p* value. Partial correlations were adjusted for the NPI score of the PwD

Figure 3 reports the principal partial correlation coefficients between the changes scores of the variables showing significant differences from pre- to post-intervention (Table 2). All change scores are expressed as improvements of the measured variable. The results indicate that greater improvement in perceived self-efficacy in dealing with patients’ disruptive behaviours is significantly related only to a greater improvement in caregiver burden levels (r = 0.386, *p* = 0.014). Concerning the other no plotted correlations, the change in caregiver burden was not significantly associated with the change in state (*r* = 0.183, *p* = 0.259) and trait (*r* = 0.246, *p* = 0.125) anxiety, and there is a significant relationship between the improvement of state and trait anxiety (*r* = 0.427, *p* = 0.006).

### Satisfaction survey

Caregivers were reported to be highly satisfied with each psychoeducation intervention session. In particular, the median score of satisfaction for Session 1 was 3 (IQR = 1), for Session 2 was 4 (1), for Session 3 was 4 (1), for Session 4 was 4 (1), for Session 5 was 3 (1), for Session 6 was 4 (1), for Session 7 was 4 (1), and for Session 8 was 4 (1). In addition, almost all the caregivers enjoyed the online course delivery method (97%). Caregivers found that the most valuable and practical information provided in the course to be the increased knowledge of the neurocognitive disease of their familiar (80.5%), the learning of strategies to manage and deal with the neurocognitive symptoms (87.8%), and the increased awareness to take time for themselves and to ask for help (48.8%).

## Discussion

This pilot study aimed to assess the effectiveness of an eHealth psychoeducation intervention designed to improve caregivers’ knowledge of the disease, the management of care recipient symptoms, the community services of practical support, and the legislation related to PwD. Results indicated the effectiveness of the intervention in improving caregiver self-efficacy in dealing with disruptive behaviours of PwD. Moreover, caregivers reported lower levels of anxiety and burden after the intervention, and the positive change in burden levels was associated with the increase in self-efficacy scores, despite the small effect size. No differences emerged for depressive symptoms and cortisol levels over time. Caregivers found the course topics and delivery mode satisfactory, as indirectly confirmed also by the low dropout rate (i.e., only one dropout due to the death of the caregiving recipient). These results provide preliminary evidence for the proposed eHealth psychoeducation intervention, highlighting the usefulness of acting on knowledge of the disease and the associated non-cognitive symptoms to increase the psychological well-being of the caregivers of PwD.

Considering the psychological outcomes of our first aim, the eHealth psychoeducation intervention improved caregivers’ burden and anxiety levels. Moreover, despite the positive impact of the intervention on caregivers’ self-efficacy scales, only the subscale related to dealing with disruptive behaviours of patients statistically improved. However, there was no effect on depressive symptoms. A recent meta-analysis has reported mixed beneficial effects of eHealth psychoeducation interventions that mainly depend on the sample characteristics or the specific topics of the course [14]. Considering similar eHealth psychoeducation interventions, the online version of the Savvy Caregiver Program found a significant reduction in caregiver burden and depressive symptoms and increased caregiver mastery and competencies [24]. Moreover, another similar eHealth psychoeducation intervention designed to promote an understanding of dementia and practical/emotional management and response to symptoms showed benefits for caregivers’ general mental health, depression, and sense of competence [50]. Therefore, our results are largely aligned with the positive effects of eHealth interventions that were mainly focused on increasing caregivers’ knowledge of dementia. The no improvement of depressive symptoms in our study could be due to a potential floor effect in the levels of depression before the intervention that could prevent the identification of changes in this variable at the end of the intervention.

Concerning the neuroendocrinological outcome of our first aim, cortisol levels remained stable in our sample from pre- to post-intervention. Thus, despite the positive effects of the eHealth psychoeducation intervention on the self-reported measure of stress, there was not a concurrent reduction in cortisol levels. One of the main challenges in using salivary cortisol as a stress biomarker is represented by the large number of psychosocial and momentary conditions that could influence its 1-day concentration and trend [51]. Moreover, previous studies have observed that psychoeducation interventions using cortisol concentration as an outcome could lead to mixed results, with an adaptive change [52] or no difference [53, 54] in cortisol levels from pre- to post-intervention. Therefore, the lack of evidence in our study is not unsurprising and does not exclude the use of cortisol levels as a biomarker of improvement in stress response mechanisms related to the attendance of a psychoeducation intervention. Indeed, recent studies using more controlled collection protocols (i.e., daily home-based collection of saliva samples for multiple days in a row) found the utility of cortisol parameters as an intervention outcome [55, 56].

Considering our second aim, the finding of an association between the increase in caregivers’ self-efficacy and the improvement of their burden levels could indicate the practical value of the proposed eHealth psychoeducation intervention. The effectiveness of psychoeducation interventions on caregivers was related to common unmet needs for knowledge about the neurocognitive disease affecting the care recipients and the management of their symptoms [57]. Indeed, ‘traditional’ or eHealth psychoeducation interventions in dementia are often structured to provide theoretical and practical knowledge that could improve self-confidence, mastery, and self-efficacy in taking care of the PwD, improving their psychological well-being [14, 58]. In particular, self-efficacy is a crucial aspect of a distressing caregiving experience [59, 60]. Indeed, literature reported that higher caregiver self-efficacy could lead to better mental health in caregivers [59, 61]. Our finding was similar to evidence found in the context of the online version of the Savvy Caregiver Program, with greater improvements in caregiver sense of competence associated with a higher reduction in caregiver burden levels [62]. Therefore, this study supports the central role of working on caregivers’ perceived confidence or self-efficacy to mitigate the caregiving deleterious effects on psychological well-being.

This study has some limitations. First, no control group was used. The pilot study was primarily designed to understand the utility and feasibility of this specific eHealth psychoeducation intervention in an Italian caregiving population. The preliminary results are noteworthy and promising for future trials that need to include a control condition. Second, the study was carried out during the COVID-19 pandemic, partially affecting the generalizability of the study. Further trials are needed during the non-pandemic period. Third, there are a few issues related to cortisol collection. For instance, the timing of saliva sampling was self-reported, and the sample collection was entirely managed by the caregiver at home. Despite the provision of clear instructions on the mode and timing of sample collection, inaccuracies are possible. Moreover, due to the pandemic condition, the pickup of the saliva samples from the caregiver’s home was, in some cases, delayed, undermining the integrity of the sample. However, the implementation of cortisol assessment in a psychoeducation intervention on caregivers during the COVID-19 pandemic remains a novelty point of our study, enriching the literature about using this parameter in the caregiver population and as an intervention outcome. Another limitation is that only one follow-up immediately after the end of the intervention was conducted, making it difficult to determine whether the intervention has long-term effects on psychological well-being and cortisol levels.

The study has some strengths, as well. First, the satisfactory questionnaire supports the positive findings of self-reported scales on the effectiveness and utility of the eHealth psychoeducation intervention. Beyond the improvement of self-efficacy scores and the reduction of stress levels, caregivers reported high satisfaction with the intervention and indicated the more valuable and practical information provided in the course the sessions related to increasing knowledge on and learning strategies to deal with neurocognitive symptoms of the PwD. Second, the study showed the feasibility of the proposed eHealth psychoeducation intervention, with a very high acceptance of the online delivery mode and a low dropout rate (2.4%). A review reported that in half of the considered studies, the completion rate for psychosocial interventions on caregivers of PwD was lower than 80% [63] and the dropout rate considering only psychoeducation interventions was 16.1% [45]. These data suggested the excellent acceptability and feasibility of the proposed eHealth psychoeducational intervention. Third, the study contributes to the need to develop and implement digital health solutions to increase caregivers’ access to training and support worldwide [1]. The average positive results of our eHealth intervention trial in the Italian context could be a good starting point for more controlled replications during the non-pandemic period.

## Conclusion

The present study has shown the positive effects of an eHealth psychoeducation intervention on self-efficacy and psychological well-being (i.e., anxiety and burden levels) in a group of Italian informal caregivers of PwD during the COVID-19 pandemic. Importantly, the improvement of caregiver self-efficacy in dealing with the disruptive behaviour of the PwD may be in part related to the decrease of caregiver burden from pre- to post-intervention. The feasibility, effectiveness, and acceptability of our intervention suggest that the proposed eHealth psychoeducation intervention could be a valuable and cost-efficient program to practically and emotionally support caregivers of PwD. Future studies with placebo and larger samples are needed to confirm the benefits of the proposed intervention.

### Supplementary Information

Below is the link to the electronic supplementary material.

Supplementary file1 (DOCX 14 KB)

## Data Availability

The dataset generated and analysed during the current study is available on Mendeley Data (10.17632/8z3n9rkv2v.1).
