# Supplementary material for: Psychobiological effects of an eHealth psychoeducational intervention to informal caregivers of persons with dementia: a pilot study during the COVID-19 pandemic in Italy
Source: Aging Clin Exp Res. 2023 Nov 9;35(12):3085–96. doi: 10.1007/s40520-023-02610-9 (PMC10721699; doi:10.1007/s40520-023-02610-9)
Supplement: Supplementary file 1 — Supplementary file1 (DOCX 14 KB) [file 40520_2023_2610_MOESM1_ESM.docx]

**Table S1.** Intervention topics.

| Session | Topic | Speaker |
| --- | --- | --- |
| Session 1 | Dementia symptoms, progression and treatments (available drugs and non-pharmacological approaches) | Geriatrician |
| Session 2 | The psychological impact and the hardship of being a caregiver | Clinical psychologist |
| Session 3 | Strategies for managing cognitive and behavioural symptoms | Neuropsychologist |
| Session 4 | Description of available services to support caregivers and patients | Social care |
| Session 5 | Legal advice | Lawyer |
| Session 6 | Strategies for managing and engaging patients at home | Occupational therapist |
| Session 7 | Caregiver stress and coping strategies | Clinical psychologist |
| Session 8 | Family associations as social support sources | Speakers from local organisations |
